# Outstanding Reviewers for *RSC Advances* in 2019

**DOI:** 10.1039/d0ra90038a

**Published:** 2020-05-19

## Abstract

We would like to take this opportunity to highlight the Outstanding Reviewers for *RSC Advances* in 2019, as selected by the editorial team for their significant contribution to the journal.
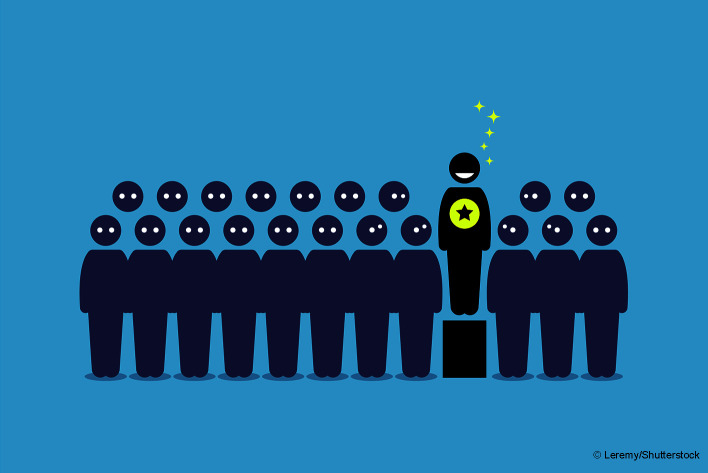

We would like to take this opportunity to thank all of *RSC Advances*’ reviewers, and in particular highlight the Outstanding Reviewers for the journal in 2019, as selected by the editorial team, for their significant contribution to *RSC Advances*. We would also like to direct a special thanks to the members of the *RSC Advances* Reviewer Panel for their hard work and dedication, and the valuable contribution they have made to the journal. The Reviewer Panel is a key part of our commitment to deliver rigorous and fair peer review and ensures that manuscripts are handled by experts throughout the peer review process. We are proud to work with these individuals and recognise their crucial role for the journal.

We announce our Outstanding Reviewers annually and each receives a certificate to give recognition for their contribution. The reviewers have been chosen based on the number, timeliness and quality of the reports completed over the year.

 

***RSC Advances* 2019 Outstanding Reviewers:**

 

Dr Arghya Adhikary

University of Calcutta

 

Professor Katsuhiko Ariga

Busshitsu Zairyo Kenkyu Kiko

ORCID: 0000-0002-2445-2955

 

Dr Yaocai Bai

University of California Riverside

ORCID: 0000-0003-3700-2520

 

Dr Xi Chen

Shanghai Jiao Tong University

 

Dr Anindita Das

Indian Association for the Cultivation of Science (IACS)

ORCID: 0000-0001-8723-6291

 

Dr Nilesh Gaikwad

Gaikwad Steroidomics Lab LLC

ORCID: 0000-0002-4990-4508

 

Professor Wei-Min He

Hunan University of Science and Engineering

ORCID: 0000-0002-9481-6697

 

Dr Mark Honey

University of Greenwich

ORCID: 0000-0001-7272-476X

 

Dr Dattatray Late

National Chemical Laboratory CSIR

ORCID: 0000-0003-3007-7220

 

Dr Giuseppe Lazzara

University of Palermo

ORCID: 0000-0003-1953-5817

 

Dr Samir Nuseibeh

University College London

ORCID: 0000-0003-1787-636X

 

Dr Veli Ozalp

Konya Gida ve Tarim Universitesi

 

Dr Qingsen Shang

University of Michigan

ORCID: 0000-0002-6782-3437

 

Dr Diptesh Sil

Atul Ltd

ORCID: 0000-0001-6457-0545

 

Dr Steven Suib

University of Connecticut

 

Dr Balaram Takale

University of California Santa Barbara

 

Dr Xiaobin Wu

Shanghai Normal University

 

Dr Murat Yavuz

Dicle Universitesi

ORCID: 0000-0003-3452-8551

 

Professor Wenwu Zhong

Taizhou University

 

***RSC Advances* Reviewer Panel 2019 Outstanding Reviewers:**

 

Dr Vipul Agarwal

University of New South Wales

ORCID: 0000-0002-6239-5410

 

Dr Ashootosh Ambade

National Chemical Laboratory CSIR

ORCID: 0000-0003-3605-5719

 

Dr Rok Borstnar

Kemijski institute

ORCID: 0000-0002-6786-5434

 

Professor Lingxin Chen

Chinese Academy of Sciences

ORCID: 0000-0002-3764-3515

 

Dr Ummadisetti Chinnarajesh

Indiana University Bloomington

ORCID: 0000-0002-0065-2223

 

Dr Emanuele Curotto

Arcadia University

 

Dr Serap Evran

Ege Universitesi

ORCID: 0000-0001-6676-4888

 

Dr Nicholas Geitner

Duke University

ORCID: 0000-0003-4313-372X

 

Dr Prokopios Georgopanos

Helmholtz-Zentrum Geesthacht Zentrum fur Materialforschung und Kustenforschung

ORCID: 0000-0002-6394-0628

 

Dr S. Girish Kumar

CMR University

ORCID: 0000-0001-9132-1202

 

Dr Hu Li

Guizhou University

ORCID: 0000-0003-3604-9271

 

Dr Jianmin Li

Zhejiang University

ORCID: 0000-0002-3917-8653

 

Dr Shiwei Qu

Scripps Research Institute

ORCID: 0000-0002-9358-066X

 

Dr Leo Small

Sandia National Laboratories

ORCID: 0000-0003-0404-6287

 

Professor David Thompson

Sam Houston State University

ORCID: 0000-0002-2934-5729

 

Dr Maria Timofeeva

FGBUN Institut kataliza im G. K. Boreskova Sibirskogo otdelenia Rossijskoj akademii nauk

 

Dr Paul Trippier

University of Nebraska Medical Center

ORCID: 0000-0002-4947-5782

 

Dr Mark Waterland

Massey University

ORCID: 0000-0002-8493-9407

 

Professor Chunping Yang

Hunan University

ORCID: 0000-0003-3987-2722

 

Professor Lei Yu

Yangzhou University

 

We would also like to thank the *RSC Advances* Editorial Board, Advisory Board and the research community for their continued support of the journal, as authors, reviewers and readers.

 

Laura Fisher, Executive Editor

Russell Cox, Editor-in-Chief

## Supplementary Material

